# Current Advances in Spinal Diseases of the Elderly: Introduction to the Special Issue

**DOI:** 10.3390/jcm10153298

**Published:** 2021-07-26

**Authors:** Takashi Hirai, Masashi Uehara, Masayuki Miyagi, Shinji Takahashi, Hiroaki Nakashima

**Affiliations:** 1 Department of Orthopaedic Surgery, Tokyo Medical and Dental University, Tokyo 113-8519, Japan; 2 Department of Orthopaedic Surgery, Shinshu University School of Medicine, Nagano 390-8621, Japan; masashi_u560613@yahoo.co.jp; 3 Department of Orthopaedic Surgery, Kitasato University School of Medicine, Kanagawa 252-0375, Japan; masayuki008@aol.com; 4 Department of Orthopedic Surgery, Osaka City University Graduate School of Medicine, Osaka 545-8585, Japan; shinji@med.osaka-cu.ac.jp; 5 Department of Orthopedic Surgery, Nagoya University Graduate School of Medicine, Aichi 466-8550, Japan; hirospine@med.nagoya-u.ac.jp

Spine-related disorders often impair quality of life (QOL) and the ability to perform activities of daily living and are a problem in rapidly aging societies. Motor deficits, poor balance, and neuropathic pain are known to be major causes of frailty in the elderly population. Spine surgeons and neurosurgeons must interpret these pathologies accurately to make a correct diagnosis and aim for an optimal solution when treating such patients. Therefore, they must be able to recognize the spinal diseases that lead to severe disorders in the elderly. This Special Issue of the *Journal of Clinical Medicine* is dedicated to current topics in spine-related disorders of the elderly (Figure 1). This narrative review focuses on present perspectives and future directions concerning refractory spinal disorders, including spinal tumors, osteoporosis, spinal metastasis, spinal deformity, and ossification of the spinal ligaments.

## 1. Spinal Deformity

In 1994, Dubousset et al. [1] introduced the “cone of economy” concept, which has since been applied when evaluating balance in elderly patients with spinal deformity. According to this concept, when the gravity line is within the cone of economy, minimal muscle activation is needed to maintain balance, but in patients with severe spinal deformity, the gravity line is outside the cone of economy, such that greater muscle energy is required and maintaining a standing posture becomes difficult [2]. Patients with spinal malalignment often complain of a variety of symptoms, including intermittent claudication due to severe low back pain, gastroesophageal reflux disease caused by excessive pressure on the abdomen due to spinal kyphosis, and depression and the resulting deterioration of QOL [3]. Schwab et al. reported that global spinal sagittal malalignment, high pelvic tilt, and spinopelvic alignment mismatch lead to the deterioration of health-related QOL in elderly patients with spinal deformity [4] and devised the well-known SRS-Schwab classification of adult spinal deformity. Recent improvements in spinal instrumentation and surgical strategies have meant that surgeons are better able to correct spinal alignment and perform long spinal fusion surgery to improve low back pain, gait, and health-related QOL [5]. However, surgery for spinal deformity has a high complication rate [6], and there are some concerns about performing highly invasive surgery in elderly patients. Therefore, we should also focus on intervention in the early stages of spinal deformity.

One of the most important factors in spinal deformity is vertebral fractures, which are prevalent in the elderly because of osteoporosis. Vertebral fractures contribute to spinal malalignment [7], and sagittal spinal malalignment is a potential risk factor for new vertebral fractures in patients with osteoporosis [8]. Therefore, treatment of osteoporosis to avoid vertebral fractures is important in terms of preserving spinal alignment.

Another frequently encountered cause of spinal deformity is the age-related decrease in muscle mass, known as sarcopenia. In a study by Eguchi et al., the incidence of sarcopenia was significantly higher in patients with spinal deformity than in those with lumbar spinal stenosis [9]. Moreover, strong correlations were found between decreased trunk muscle mass, global spinal sagittal malalignment, and poor low back pain, and health-related QOL scores [10]. These findings indicate that the measurement of skeletal muscle mass is required for a diagnosis of sarcopenia. However, the measurement of trunk muscle mass may also be needed in patients with spinal deformity. Furthermore, the management of spinal deformity should include early intervention to improve both skeletal and trunk muscle mass.

## 2. Osteoporotic Vertebral Fractures

Osteoporotic fractures are particularly common in vertebrae, and their diagnosis and treatment are very important in our aging society. An osteoporotic vertebral fracture (OVF) not only reduces QOL by causing chronic low back pain and a decrease physical activity but also increases the mortality risk by about 15% [11]. Although an OVF is not difficult to diagnose, there are some points that need to be taken into account so that these fractures are not missed or misdiagnosed. Considering that the risk of a future OVF is markedly increased in patients with a prior OVF, the correct diagnosis and treatment of osteoporosis are important to reduce the risk of a future fracture. The first 1–2 years after OVF is a time of increased risk for further fractures and is known as the imminent fracture risk period [12]. Therefore, therapeutic intervention for osteoporosis should be initiated promptly at the time of an OVF. The goals of treatment are pain relief and the achievement of the pre-injury QOL and activities of daily living (ADL) performance level. Although conservative measures are the mainstay of treatment for OVF, there is still no gold standard approach in terms of bracing and physical therapy. The choice of treatment must be tailored to the patient’s general condition and previous level of physical activity.

At present, there is no OVF classification that can be used to guide treatment. Although the Genant classification [13] is the most widely used in epidemiological and clinical studies because of its simplicity, it does not categorize OVF according to morphology, making it difficult to use for treatment decisions. More recently, the German Society for Orthopaedics and Trauma has proposed a classification that can be used to decide feasible treatment [14,15]. However, in elderly patients, the treatment goals will vary greatly depending on overall health status and the pre-fracture ability to perform ADL, so the extent to which this scoring system is actually useful needs further validation.

It is well known that elderly patients are at high risk of surgical complications. Vertebral augmentation, posterior fixation, osteotomy, and anterior fixation are the surgical options for OVF. It is necessary to understand the advantages and disadvantages of these techniques before selecting a surgical method. Vertebral augmentation or local fixation is indicated when the aim is to reduce back pain caused by a local fracture or pseudoarthrosis, whereas decompression fixation is indicated for neurological symptoms. However, there are some potential problems with implant surgery, such as loss of correction, adjacent vertebral fracture, leakage and migration of cement, and a need for revision surgery [16,17]. Preoperative imaging evaluation is important to minimize the risk of these complications. 

The risk of complications with implant surgery increases in patients over 80 years of age [18]. Posterior fusion is indicated when the vertebral body shows mobility but is judged to be poorly improved by vertebral augmentation alone based on imaging findings or when neurological symptoms are present. If the mobility of the vertebral body is observed, vertebral body augmentation can be used to support the anterior column. However, the correction effect is poor [19]. Loss of correction after posterior fusion is caused by loosening of the screw on either the cephalad or caudal side. For posterior fixation, the pedicle screw alone may pull out, and a combination of various hook systems and wire ring taping should be considered. Augmentation of fenestrated pedicle screws is also an option but has been reported to have a high cement leak rate [18].

Anterior column reconstruction is necessary when the fractured vertebral body is unstable but not amenable to vertebral augmentation, when there is instability at the disc level, and when there is prominent vertebral body deformation that requires resection. The anterior column can be reconstructed by a posterior osteotomy or anterior discectomy, but posterior anchoring is essential for the correction of kyphosis. According to one report [20], an operating time of 3 h and blood loss of less than 500 mL is acceptable in patients over 80 years of age. Therefore, the anterior reconstruction can be performed if short fusion is possible but is not otherwise indicated in this age group.

OVF is basically treated conservatively in elderly patients, but surgery should be considered for those who are resistant to conservative measures and those with neurological symptoms. Given that OVF is often difficult to treat, it is important to start screening for osteoporosis at a relatively young age and initiate treatment as early as possible.

## 3. Tumors of the Spinal Cord

Spinal cord tumors are rare and account for 4–16% of all tumors in the central nervous system [21,22,23,24]. A previous study in our orthopedic department identified 678 patients with spinal cord tumors over a 10-year period. Most of the patients who required surgery were in their 50 s or 60 s, with a mean age at the time of surgery of 52.4 years.

Intramedullary spinal cord tumors (ImSCTs) comprise approximately 5–20% of all spinal cord tumors [21,22,23,24]. Although glioma and astrocytoma are common ImSCTs in pediatric patients, ependymomas are the most common ImSCT in adults. Ependymomas tend to be located in the cervical or cervicothoracic region in adults, whereas most are myxopapillary ependymomas in the conus medullaris in pediatric patients. Other ImSCTs commonly found in adults are hemangioma and hemangioblastoma [23]. Most hemangiomas are found at the cervical and thoracic levels and are typically observed in patients aged older than 50 years. Three-quarters of hemangiomas are intramedullary, and the remainder are at intradural or epidural sites. Adult hemangioblastoma is most likely to occur in those in their 30s [23]. Approximately 90% of hemangioblastomas are ImSCTs, and the remainder are intradural-extramedullary tumors. Moreover, 40% of hemangioblastomas are associated with von Hippel–Lindau disease and whole-body computed tomography is needed to confirm the diagnosis.

Intradural-extramedullary tumors are the most common spinal cord tumors. In Asian populations, most intradural-extramedullary tumors are schwannomas followed by meningiomas [23]. However, in non-Asian countries, the incidence of meningioma is at least as high as that of schwannoma. Schwannoma is typically diagnosed after the age of 30, with the highest incidence in those aged 50–59 years [23]. In contrast, meningioma typically occurs after the age of 50, and its highest incidence is among those aged 60–69 years [23].

Recent progress in research on spinal cord tumors includes advances in the genetic analysis and identification of causative genes for various tumors [21]. Meningioma is one of the most common spinal cord tumors, and the most consistent genetic abnormality found in patients with these tumors is a complete or partial loss of chromosome 22, followed by the loss of 1p, 9p, and 10q and gain of 5p and 17q [21]. Further advances are expected in the field of genetic analysis, and in addition to arranging the pathological classification of tumors, developments in gene therapy for malignant tumors are anticipated.

## 4. Spinal Metastasis

In recent years, there has been an increase in the number of patients with metastatic spine tumors due to advances in the treatment of their primary cancers and population aging. Metastatic spinal cord compression occurs in 5–14% of all patients with cancer [25]. Therefore, it is important to detect and treat compression because it may lead to pathological fractures and paralysis, both of which can greatly impair ADLs [26]. Nonetheless, many patients develop severe paralysis due to spinal cord compression and require emergency hospitalization. In such cases, improvement after surgery is poor, and the risk of complications is high. Therefore, it is necessary to establish a comprehensive treatment plan that takes into account the likely prognosis and the patient’s overall state of health.

In 2005, Patchell et al. reported a randomized trial in which they found that surgery was more effective than radiotherapy for metastatic spinal cord compression in terms of maintaining the ability to walk [25]. However, a subsequent study [27] found that the therapeutic outcome of radiation was inferior to that previously reported. Rades et al. investigated 11 prognostic factors in a cohort of 2296 patients with metastatic spinal cord compression. Using matched-pair analysis, they were able to compare the outcomes between 108 patients who underwent surgery plus radiotherapy and 216 patients who received radiation alone [28]. They found no significant difference in terms of improvement in motor function or the ambulation rate between the two groups and reported a surgery-related complication rate of 11%. They concluded that patients over 65 years of age with metastatic spinal cord compression did not benefit remarkably from the addition of surgery to radiotherapy in terms of functional outcome, local control of metastatic spinal cord compression, or survival [29]. In a systematic review of metastatic spine tumors, the incidence of complications from radiotherapy alone was unknown, and few studies had documented the progression of the systemic disease during treatment [27]. The overall surgical complication rate was 29% (range, 5–65%), and the 30-day postoperative mortality rate was 5% (range, 0–22%) [27]. The guidelines for diagnosis and treatment of bone metastasis published by the relevant Japanese societies weakly recommend surgery for functional improvement [30].

Further prospective randomized trials are needed to establish the value of surgery for metastatic spinal cord compression. In recent years, minimally invasive techniques, such as percutaneous pedicle screw fixation, have become popular and are reportedly useful in patients with high-risk metastatic spinal tumors [31,32]. Advances in surgical techniques and minimally invasive surgery are expected to improve surgical outcomes in the future.

## 5. Ossification of Spinal Ligaments

The spinal ligaments stabilize the structure of the entire spine and are known to work together to allow spinal mobility. Ossification of the spinal ligaments (OSL) is a result of heterotopic bone formation in the spine. This pathologic state is well known to be more prevalent in Asian countries than in the West. Basic studies have identified several genetic factors that are associated with the onset and extension of ossification of the posterior longitudinal ligament (OPLL). Karasugi et al. [33] performed a genome-wide linkage study based on 214 sibling pairs with OPLL and identified loci with suggestive linkage on 1p21, 2p22–2p24, 7q22, 16p24, and 20p12. Furthermore, Nakajima et al. identified six putative genes in a genome-wide association study (GWAS) that included 1130 patients with OPLL and 7125 controls [34]. Given that the GWAS could explain less than 2% of the total genetic variance of OPLL, further information is necessary to understand the mechanisms.

Current advances in diagnostic imaging technology have improved our understanding of the distribution and extension of OSL. Hirai et al. investigated the distribution of ossified lesions throughout the whole spine using low-dose radiation computed tomography (CT) and demonstrated that the prevalence of OPLL in the whole spine is correlated with female sex, body mass index, and the degree of OPLL in the cervical spine [35]. They also found a strong association between OSL and other concomitant ossified lesions in the cervical and thoracic spine [36,37,38,39,40]. Katsumi et al. evaluated the chronologic progression of ossified lesions on three-dimensional CT images for patients with OPLL. They reported a mean annual increase in the lesion rate of 4.1% in non-surgically treated patients and identified younger age and obesity to be risk factors for progression. Interestingly, they also showed that the mean annual increase in the lesion rate was significantly lower in patients who underwent posterior decompression with fusion surgery than in those who underwent laminoplasty without fusion (2.0% vs. 7.5%). Considering that OPLL often results in the onset and deterioration of spinal disorders with increasing size, it is important for physicians and spine surgeons to conduct future investigations to identify significant predictors of extension and thickening of ossification.

Clinically, surgery is indicated in patients with cervical OPLL who have progressive myelopathic symptoms, such as clumsy hands, a spastic gait, and bowel or bladder impairment that lead to severe restriction of everyday activities. Two major surgical strategies, namely, laminoplasty and anterior decompression with fusion, have been used in patients with OPLL and myelopathy. Laminoplasty was developed in Japan as a minimally invasive and effective posterior strategy that is relatively easy and safe to perform [41] and is now used throughout the world. However, despite these advantages, laminoplasty is not suitable for patients with extensive ossified lesions (with a canal occupying ratio > 60%), beak-type OPLL, or cervical kyphotic alignment because of decompression at the ventral portion of the spinal cord [42,43]. The anterior technique essentially decompresses the ossification and stabilizes the structures of the cervical spine. Compared with laminoplasty, anterior corpectomy with fusion achieves better clinical outcomes in patients with extensive OPLL and/or kyphotic alignment [44]. Posterior decompression with fusion is a further strategy that can be used to achieve decompression and stability in these patients with outcomes similar to those achieved using the anterior method [45]. However, to obtain a good clinical outcome, it is best to operate on a patient with OPLL before the spinal cord is damaged irreversibly. We, therefore, should weigh the benefits of these three types of surgery in patients with a spinal disorder caused by ossified lesions.

## Figures and Tables

**Figure 1:**
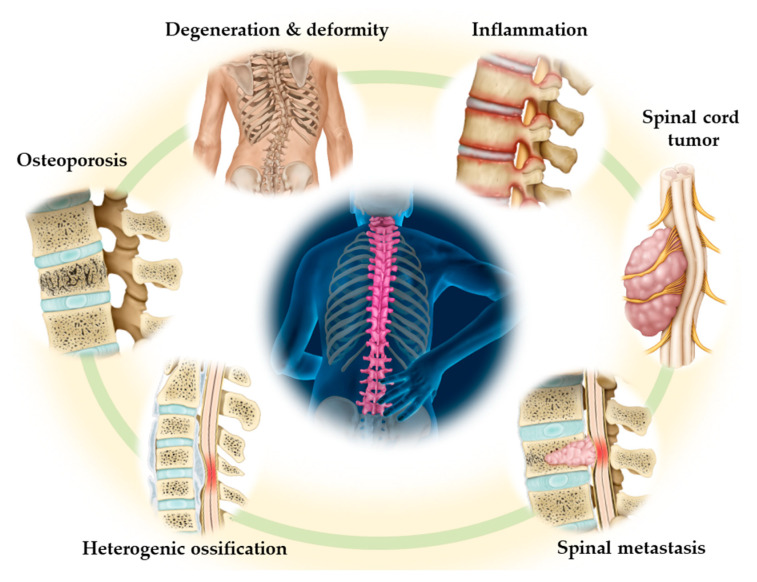
Typical spine-related disorders in the elderly.
